# Social Support in a Cancer Patient-Informal Caregiver Dyad: A Scoping Review

**DOI:** 10.3390/cancers15061754

**Published:** 2023-03-14

**Authors:** Małgorzata Pasek, Anna Goździalska, Małgorzata Jochymek, Rosario Caruso

**Affiliations:** 1 Department of Nursing, Faculty of Health, University of Applied Sciences in Tarnów, 33-100 Tarnów, Poland; 2 Faculty of Health and Medical Studies, A. F. Modrzewski Krakow University, 30-705 Krakow, Poland; 3 Health Professions Research and Development Unit, IRCCS Policlinico San Donato, San Donato Milanese, 20097 Milan, Italy; 4 Department of Biomedical Sciences for Health, University of Milan, 20133 Milan, Italy

**Keywords:** social supports, dyads, cancer, scoping review, systematic review

## Abstract

**Simple Summary:**

In addition to studies on the quality of life, hope, self-efficacy, and unsatisfied needs, research on social support is a priority in the search for ways to cope with cancer, which affects a patient and their relatives. Coping with cancer, therefore, also applies to caregivers. Hence, psychosocial interventions that reduce the level of stress and, above all, improve the ability to cope with difficult situations are more effective when conducted in dyads. A scoping review of studies on the impact of social support in a dyadic patient-informal caregiver relationship during cancer treatment in the period 2012–2022 was conducted. Thirteen articles met the inclusion criteria and qualified for the analysis.

**Abstract:**

Social support that includes promoting healthy behaviours throughout the oncology pathway, from diagnosis to treatment to survival, can leverage existing support networks and improve the health of patients and family members in supportive roles. This scoping review aimed to identify and summarise the impact of social support on the patient-informal caregiver relationship during cancer treatment. Inclusion criteria were related to a high focus on dyadic cancer patient-informal caregiver relationships, considering a population of adult cancer patients in active hospitalisation on an oncology ward, and published between 2012 and 2022 to get a portrait of the literature that might influence the current practice. A systematic search using the “Population, Concept, and Context” framework was performed in PubMed, Web of Science, SCOPUS, EBSCO Medline, and CINAHL: 13 articles from the 16,425 pre-qualified articles published between 2012 and 2022. The narrative synthesis of the included studies highlighted that social support, encompassing its different forms within the context of dyads, is frequently associated with an enhanced quality of life, hope, and resilience of both patients and informal caregivers. However, it is important to recognize that the support interventions provided to patients, particularly caregivers, were frequently not thoroughly evaluated or explained, and the sample sizes of the included studies were often limited. Therefore, this review clarified the social and clinical potential of social support for the patient-informal caregiver relationship, paving the way for future robust studies that require to be powered and designed on specific outcomes to allow informing the practice on specific recommendations.

## 1. Introduction

Research on social support is a crucial aspect of coping with cancer, alongside studies on quality of life, hope, self-efficacy, and unsatisfied needs. Cancer affects not only the patient but also their closest person and the family as a whole, making social support particularly important. In fact, cancer often impacts dyads, such as couples, as an interdependent system, leading them to respond to the illness as a unit rather than as separate individuals [1]. As a result, psychosocial interventions that reduce stress and improve coping abilities are most effective when implemented in dyads [2,3,4,5].

A patient-informal caregiver dyad is a pair consisting of a person diagnosed with cancer (the patient) and their closest non-professional caregiver (the informal caregiver). This relationship is important as cancer affects not only the patient but also their caregiver and family. The term “informal” distinguishes the caregiver in this context from professional caregivers who provide medical care. Informal caregivers are usually family members or friends who provide emotional and practical support to the patient during their illness. A patient-informal caregiver dyad is a crucial unit of care in cancer management, as both individuals are interdependent, and their social functioning is often disrupted by cancer diagnosis and treatment. Cancer can significantly affect their mutual interactions and relationship requirements, leading to changes in social functioning. For instance, a study conducted on dyads, precisely on couples where the women had breast cancer, showed that they prioritised their own needs over their partner’s and the relationship, which highlights the need to reformulate the dyadic relationship to help couples maintain proper relations during early survival while considering the need for additional support and resources. It is essential to emphasise that social support should be provided to dyads at the right time, along with targeted resources, to ensure that the patient-informal caregiver dyad receives appropriate care and support [6].

Social support in cancer care refers to the range of assistance and encouragement provided by individuals or groups within a patient’s social network [7]. This includes emotional support, informational support, and other types of support that promote healthy behaviours at various stages of the oncological path, from diagnosis to treatment to survival. Utilising existing support networks can improve the health of both patients and their supportive caregivers [7]. Social support has been shown to have a moderating effect and can positively impact psychological functioning even after the stressor subsides [8]. Caregivers who provide emotional and informational support can significantly contribute to patient activation, leading to greater participation in treatment and adherence to prescribed regimens [9].

Research conducted by Pasek et al. on the assessment and need for social support in patient-caregiver dyads found that patients perceived receiving more support than their informal caregivers [10]. This result could suggest that caregivers received less support from medical professionals than patients did [10]. However, studies by Regan et al. showed that patients and their partners had different views from health professionals regarding their psychosocial needs, and professionals from various disciplines also had divergent views about the dyads’ psychosocial needs. Although most physicians believed that dyad-centered psychosocial care was necessary, most of the dyads surveyed did not see the need for specialist support and intervention focused on them. Thus, the need for social support and intervention for cancer dyads may be underestimated or not fully appreciated; it is essential to ensure that they receive the appropriate support and resources at the right time [4].

Measuring the need for support and its targeted provision may contribute to defining research protocols and useful management strategies to improve the general health of cancer patients and their informal caregivers [11]. Some national institutions point to the importance of maintaining a patient-caregiver dyad for improving the effects of cancer treatment and creating strategies for integrating caregivers with formal healthcare environments [12].

When examining different aspects of how dyads function in facing the challenges of cancer care, it was observed that patients and informal caregivers who had a high degree of insecure attachment tended to experience lower levels of social support and higher levels of depression and/or anxiety symptoms [13]. A common understanding of one’s own needs in a dyad is the reason for asking for support. Informal and formal social networks are also helpful in providing support in various aspects, thus avoiding experiences of isolation and helplessness in the face of disease. Psychosocial support has been defined as the basic dimension that helps a dyad maintain a positive relationship in their dyad in both the acute phase of treatment and the early phase of survival [14].

Thus far, the recent literature on this topic was not recently summarised, undermining the possibility of researchers, clinicians, and educators to identify the current state of knowledge and the gaps in research regarding social support in a cancer patient-informal caregiver dyad. In other words, the lack of literature reviews in this regard may limit the ability to draw meaningful conclusions in a decision-making process or develop evidence-based interventions to improve social support for patients and their informal caregivers. For these reasons, the aim of our study was to conduct a scoping review of the available research on the impact of social support in the dyadic relationship between a patient and their closest person, i.e., informal caregiver, during cancer treatment.

## 2. Materials and Methods

This study was a scoping review [15], and the Preferred Reporting Items for Systematic Reviews and Meta-Analyses (PRISMA) statement (extension for scoping reviews) [16] guided the study and its reporting [16,17,18] (see Supplementary File). The systematic searches were based on the population/concept/context (PCC) framework [19]. In the queries, the population was defined as cancer and neoplasms; the concept concerned relationships in dyads that are defined as an informal caregiver’s contribution and interaction with a patient. The context covered social support.

Due to its scoping nature, the study protocol did not qualify for registration in the PROSPERO database, even if scoping reviews are performed within the framework of systematic searchers [20].

The research project was approved by the Bioethics Committee of the Andrzej Frycz Modrzewski Krakow University (Resolution 45/2022 of the Bioethics Committee of the Andrzej Frycz Modrzewski Krakow University of 22 September 2022 on issuing opinion no. KBKA45/O/2022).

### 2.1. Search Process and Sources

A systematic literature search was conducted using five electronic databases: PubMed, Web of Science, SCOPUS, EBSCO Medline, and CINAHL. The search period covered the years 2012–2022. Zotero software was used to create the repository that included the identified records. We combined words from Medical Subject Headings (MeSH) and free-text words using a single-line search strategy [21]. This search strategy was designed primarily for the PubMed database, and for the purposes of our study, it was modified for the other databases. The reference lists of meaningful studies (e.g., a qualitative study) were used to identify any additional articles not identified by the queries and were compared and searched manually, as recommended by Richards [22]. In the searches, no language limitations were used for the record identification phase.

The query was performed using the following terms: (“neoplasms” OR “cancer”) AND (Care-givers OR Family OR ‘Adult Children’ OR ‘informal care’ OR caregiver OR spouse OR husband OR wife OR family OR families OR son OR daughter OR partner OR couple OR caregiver OR caregivers OR dyad*) AND (‘Social Support’ OR ‘social support’). In PubMed, the query was developed in accordance with MeSH, which is used to search for biomedical and health-related information.

Articles were selected in stages based on titles, abstracts, full texts, and inclusion and exclusion criteria. This process was always carried out by two independent authors (different authors for different databases). In the absence of an agreement to include an article, an attempt was made to obtain a consensus, and in the absence of a consensus, a decision was made by a third author not involved in the development of the given database.

### 2.2. Inclusion Criteria

Studies had to meet the following criteria to be eligible for inclusion in this review. First, the study had to focus on dyads (i.e., dyadic study), in which the patient was an adult diagnosed with cancer and was actively receiving treatment in an oncology ward. Dyadic studies have to involve collecting data from both individuals in the relationship and analysing how their interactions and behaviours impact each other. Second, the caregiver(s) included in the study had to be unpaid for their caregiving activities and defined as the patient’s closest person, such as a family member. Finally, to ensure that the review included recent and relevant evidence, studies published from 2012 to 2022 were considered for inclusion. These criteria were established based on a detailed analysis of the eligible research and were designed to identify studies that would provide valuable insights into social support in the context of cancer care.

### 2.3. Exclusion Criteria

To ensure that the findings of our review were relevant and reliable, we established clear inclusion criteria during the screening phase. Specifically, we excluded records that focused on pediatric patients, the terminal phase of cancer, or the period after oncological treatment. We also excluded records that examined the scope of social support for only caregivers or patients, as we aimed to investigate the dyadic relationship between a patient and their informal caregiver. Finally, we excluded records that focused on hematological diseases in a patient to maintain consistency in our sample.

### 2.4. Selection Flow

**Figure 1:**
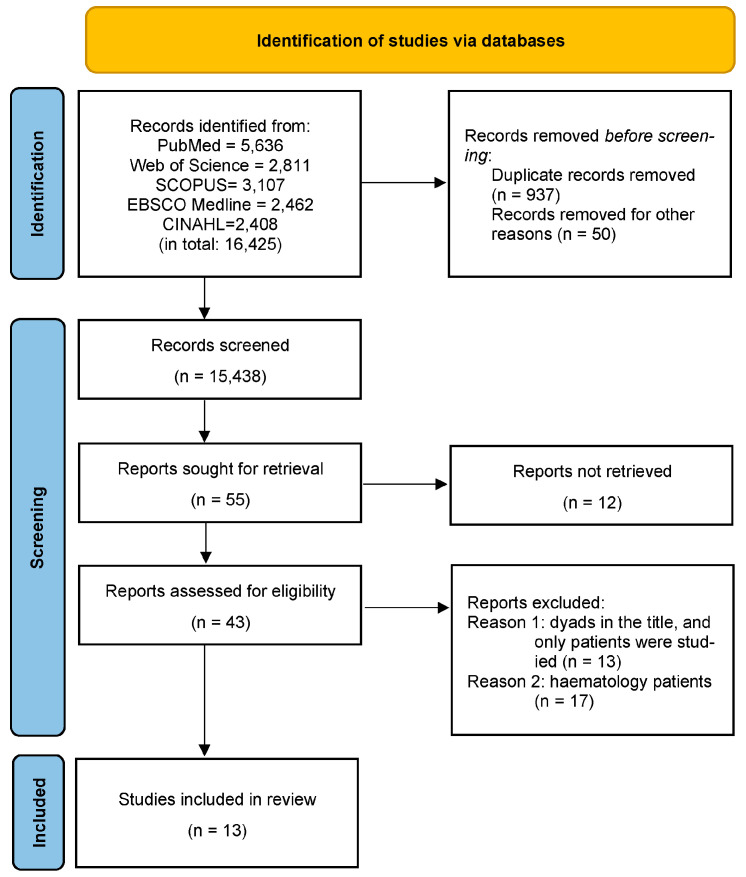
Preferred Reporting Items for Systematic Reviews Extension for Scoping Reviews Checklist (PRISMA-ScR) flow diagram. Note: In the screening phase, 15,383 records had a title/abstract not focused on a patient-informal caregiver dyad.

We conducted a literature search and identified 16,425 articles published in English. Figure 1 illustrates the article selection process. We removed 937 duplicate records and 50 records that contained only English abstracts, but the articles were in a foreign language. After screening titles and abstracts, we excluded 15,383 records that were not relevant to our study. Of the remaining 55 records, we were unable to obtain 12 (primarily conference proceedings) through our institutional library system. Of the remaining 43 studies, 13 included the term “dyad” in the title but focused solely on patients, while 17 records involved hematology patients and were excluded. Ultimately, we included 13 records in our analysis.

### 2.5. Data Extraction and Synthesis

Two independent reviewers performed data extraction using a data extraction form designed for this study, and disagreements were resolved through discussion or consulting a third reviewer. The form included information on the study design, sample size, characteristics of patients and caregivers, social support measurement tools, and key findings related to social support. After extracting data from the included studies, we performed a narrative synthesis of the findings. This synthesis involved extracting the key findings related to social support in the context of cancer care and narratively summarising these findings across the included studies. The synthesis was organised around the key concepts that emerged from the data, which included the types of social support provided by informal caregivers, the impact of social support on the caregiver and patient, and the barriers and facilitators to providing social support. The narrative synthesis allowed us to identify literature gaps and make future research recommendations.

## 3. Results

The research results refer to 13 peer-reviewed included articles examining social support in a cancer patient-informal caregiver dyad, published in 2012–2022, on two continents (Europe and North America). The articles were published in the United Kingdom (*n* = 7), the United States of America (USA) (*n* = 4), Greece (*n* = 1), and Poland (*n* = 1).

The included studies were conducted in Turkey (*n* = 3), Poland (*n* = 3), the USA (*n* = 2), South Korea (*n* = 1), Australia (*n* = 1), and China (*n* = 1), and international research was carried out in the USA/Israel (*n* = 1) and Austria/Israel (*n* = 1).

Table 1 shows the characteristics of the included studies.

The majority of studies (*n* = 12) were cross-sectional studies, and only one was a qualitative study [29] carried out using individual interviews.

Implementing research in patient-caregiver dyads, which involves collecting research material, is rather time-consuming; in this regard, seven articles contained no information, but in the remaining included studies, the duration of the psychosocial support interventions oscillated from five months [25] through more than one year [23,33] to three and more years in three studies [27,31,32].

The study site was determined by the inclusion criteria of the research and can be categorised into two main types. The first type (*n* = 7) included oncology departments that provided radiotherapy and clinical oncology/chemotherapy [10,23,26,31], hospitals, or oncology hospitals [25,27,28], where cancer patients treated with anticancer drugs were recruited. The second type consisted of wards (*n* = 6), where patients with specific clinical diagnoses were hospitalised, such as gastric cancer [29], head and neck cancer [32], colorectal cancer [30], breast cancer [24] and genital cancer [33,34]. In all cases, caregivers were individuals indicated by patients and included relatives, spouses, children, parents and unrelated persons. The purposes of the research projects varied. While social support was a key focus in all cases, it was either the primary objective or was investigated in relation to other variables. Two studies aimed to explore the level and sources of social support in dyads [26,30], and one study examined the factors influencing multidimensional social support in dyads [31]. Another study aimed to investigate the potential mediating role of perceived social support in the functioning of couples [25], while the moderating role of social support in the mental well-being of the patient and caregiver was explored in a different study [29]. Social support was also investigated in relation to quality of life [23], acceptance of illness [10], hope [27], depression [28], well-being in newly diagnosed patients [32], as well as anxiety and hopelessness [34].

The research tools used were developed adequately for the purpose of the included studies, and the included scales examining social support were most often standardised. The scales used to assess multidimensional support included the Berlin Social Support Scales [10,26,31], the Source-Specific Social Provisions Scale [24], the Cancer Perceived Agents of Social Support [27], the Cancer Perceived Agents of Social Support [28], the Duke-UNC Functional Social Support Questionnaire [29], the Patient Social Support Form and Family Social Support Form [33,34]. The second group of tools aimed to examine a specific type of support, most commonly the support received, and included the Multidimensional Scale of Perceived Social Support [23] and the Perceived Social Support Scale [25]. One of the studies used open-ended questions to elicit statements about social support [32], and a qualitative study [30] was conducted using a prepared interview scheme.

The studied group consisted of patients and caregivers. In most articles (*n* = 11), they were groups of equal size, in which the study of relationships was carried out in pairs. In two articles (*n* = 2), group sizes differed. In one case, the authors qualified all the returned questionnaires [24]. In another case, despite a different number of patients (*n* = 52) and caregivers (*n* = 36), a generalisation was used that 52 dyads were examined [29]. The size of the studied groups in the cross-sectional projects ranged from 52 to 318 dyads, with 150 and more couples recruited in *n* = 7 studies.

Following the accepted standards of statistical evaluation of the collected data, descriptive statistics were used to characterise the studied group, with inferential comparisons based on a significance level of *p* < 0.05. Statistical relationships were investigated using the Kruskal–Wallis test, ANOVA, the Pearson’s correlation test, Kendall’s tau-b coefficients of linear correlation and Brown correlation analysis. Multilevel modelling (MLM) [24], mediation [10,25], structural equation modelling (SEM) [28], hierarchical multiple regression analyses [28] and multiple regression analysis using the stepwise progressive method [31] were used.

An important aspect of our scoping review is the analysis of the type of social support studied and learning about the key findings, including the relationship in a cancer patient-caregiver dyad. Table 2 shows the detailed data.

Some of the conducted studies (*n* = 6) showed a positive and important relationship between the results of patients and their informal caregivers [10,23,24,26,27,30]. In one study, there was no dyad effect; social support for patients did not predict caregiver outcomes, nor did social support for caregivers predict patient outcomes [29]. Another study found no significant correlation between the social support provided by caregivers as perceived cancer patients and the social support that caregivers believed they provided to patients [33].

Significant and positive correlations were found between family resilience, perceived social support, and individual resilience of both patients and spouses (r = 0.13–0.57, *p* < 0.05) [25].

In addition, studies showed that cancer patients received more support than their caregivers [26].

Ayik et al. showed a statistically significant positive relationship between the perceived total social support and the overall quality of life subscale in both the patient and the caregiver. In addition, an impact on positive business results of the caregiver was found [23].

Boeding et al. pointed out a different correlation. Namely, women with breast cancer who complained about mood swings and/or fatigue reported that they received a higher level of support than women who did not experience negative mood. Women whose husbands indicated a higher level of marital satisfaction reported receiving more support from their partner, but their husbands’ marital satisfaction did not reduce the impact of women’s mood on support [24].

Chen et al. drew attention to the correlation of patients’ family resilience, perceived social support and individual resilience scores with the characteristics of their spouses (r = 0.24–0.32, *p* < 0.01). There was a significant positive effect of perceived social support by patients and spouses on patients’ individual resilience (β = 0.33, *p* = 0.000 and β = 0.47, *p* = 0.000, respectively) but not on their partners’ resilience. Spouses’ family resilience was related to their partners’ individual resilience through the total mediating effect of their partners’ perceived social support (β = 0.04, 95% CI = 0.005, 0.082) [25].

The study of support and hope showed a positive correlation among patients and spouses between perceived social support and their own level of hope (β = 0.44, *p* < 0.0001, β = 0.56, *p* < 0.0001) and a negative correlation with the level of hope in the other person (β = −0.25, *p* < 0.024; β = −0.44, *p* < 0.0001) [27]. Uslu-Sahan et al. proved that patients felt greater hopelessness and fear of death compared to caregivers. Social support had a statistically significant effect on the sense of hope or the lack thereof in 35% of patients and in 40% of caregivers [34].

Depression was also found to be more common in older patients and their spouses than in younger patients. Specifically, older patients and spouses reported lower levels of depression than younger patients. The structural equation model showed that social support was directly related to depression in younger women and older spouses, while hope was directly related to depression among older women and younger spouses, and acted as a mediator between social support and depression [28].

In the study by Jeong et al. [29], social support for patients was a predictor of their anxiety, and it explained both depression and anxiety in caregivers. In addition, it was shown that the income level of patients made it possible to significantly predict their anxiety [29].

In the qualitative study, respondents indicated various dimensions of support; the importance of shared experience; and of practical, financial, and emotional support. The treatment team played an important supportive role [30].

In the study of detailed support for patients and their caregivers, no statistically significant difference was noticed in the perceived emotional, information, and instrumental support, and the need for support and seeking support were on medium levels in both groups. On the scale of support, statistically significant differences between the examined patients and their caregivers occurred for the support currently received (*p* < 0.01), emotional support (*p* < 0.05), and the general level of protective buffering support received (*p* < 0.001). There was a statistically significant correlation between information support in the group of caregivers and the need for support (0.23), and the sense of support (0.16) in the group of patients. A positive correlation was obtained, indicating that the need for support and the sense of support in patients soared along with an increase in information support for caregivers [31].

Perceived social support in dyads was high, but the values of this parameter in cancer patients proved to be higher than in their caregivers. In both patients (t = −3.82; *p* < 0.001; d = 0.40) and their caregivers (t = −2.25; *p* = 0.027; d = 0.21), the level of perceived instrumental support was higher than the level of perceived emotional support. This indicates the important role of the interaction between a patient and their relatives as a determinant of acceptance of the disease. While experiencing the disease, a patient and their relatives formulate and modify their opinions about cancer, its course, and its consequences. These opinions, defined as the image of one’s disease, are largely determined by the sense of coherence [10]. The study by Suruku et al. showed that the perceived social support for cancer patients provided by their caregivers is higher than the level of support given to patients, as assessed by caregivers [33].

On the other hand, in the study by Sterby et al., emotional support, with particular emphasis on spiritual support, was most often expected by both patients and caregivers [32]. Caregivers most often reported that they received emotional and instrumental support, with an emphasis on nutrition and help in terms of speech, appearance and coping with addictions. While patients experienced greater physical strain than caregivers, they faced emotional challenges just as much. Caregivers also emphasised more often than patients that they received critical instrumental support, including help with finances, transportation to appointments, cooking and other household chores [32].

## 4. Discussion

The aim of this study was to map the impact of social support on the patient-informal caregiver relationship during cancer treatment. We conducted a search on the PubMed database using a search strategy based on individual MeSH terms, which we adapted for other searched databases. Our search yielded 16,425 articles, reflecting the broad definition of the term ‘cancer’. While the MeSH dictionary did not provide a direct definition of a dyad, it indirectly described it. After applying our inclusion and exclusion criteria, we retrieved and thoroughly analysed 13 articles representing the most recent knowledge on this topic. The emerging results mapping the recent literature on the investigated topic can potentially inform practice and research in this area.

More precisely, the results indicate that social support can influence various aspects of the dyadic relationship, including general well-being, medical interaction, sexual function, symptoms and physical activity, social relationships, business performance and overall quality of life [10,23,25,26,27,28,31]. Adequate social support should be tailored to a patient’s needs, as excessive support may negatively impact their activity and independence. The study also highlights the importance of early intervention for patients and caregivers to prevent the subsequent development of mental stress in the dyad [31]. Longitudinal studies can be planned in the future to determine the changing picture of social support at various stages of cancer treatment.

Research on coping with cancer indicates that not only a patient but their caregiver is also affected by cancer, which is why couples may react as individuals, not as separate individuals [1]. In all studies conducted in oncology departments and hospitals, where the sampling criterion was cancer [10,23,25,26,27,28,31] and, additionally, a diagnosis of breast cancer was present [24], a dyadic relationship was confirmed in the context of social support. For patients, the subjective sense of support during treatment depends on the need for help shown to their caregivers [31].

Two studies in which a patient had a specific type of cancer consisted of gastric cancer [29] and reproductive organ cancer [33], which showed no dyad effect or no significant correlation between perceived social support in the dyad. This aspect may reflect the small size of the groups (52 and 69 dyads, respectively). One may also wonder whether the presence of patients with a specific cancer location in the project does not require the use of more specific research tools related to, for example, the bio–psycho–social impact of functioning with cancer. The importance of searching for various relationships is confirmed by psychological research, which shows that early intervention for a patient and their caregiver as well as proper social support, can prevent the subsequent development of mental stress in the dyad [35].

Research has also shown that cancer patients received more support than their caregivers [26], although patients’ need for support was lower. Social support should be adequate for a patient’s needs to enable them to develop optimal methods of coping with the disease. Excessive support and participation in a person’s life may negatively impact their activity and cause the loss of independence and the inability to consciously use internal and external resources to cope with a long-term stressful situation [10].

The review of eligible articles has shown that social support can influence various aspects. A study of women with breast cancer showed a relationship between a higher level of their positive mood and the support they received from men. At the same time, receiving more support may be the basis for the patient to believe that her health condition requires such a level of support, which may intensify her negative feelings [24].

In the case of the correlation of support with resilience, the full mediation effect was not obtained in the spouses. The perceived social support of patients and spouses positively affected their individual resilience, but not their mutual resilience [25]. There was a relationship between perceived social support and general well-being, medical interaction, sexual function, symptoms and physical activity, social relationships and business performance, and overall medium quality of life scores.

The support received by patients and provided by caregivers is inadequately assessed as greater or lesser [10,26,33]. These results can be used in medical and psychological practice. They allow for targeted and conscious work with emotions, for example, in the cognitive-behavioural approach.

It has been observed that the perceptual definition of social support made it possible to highlight important aspects of this concept, the level of support obtained from various sources and the degree of satisfaction that dyad members feel with this support. It is also particularly beneficial in research on the buffering effect of stress on people’s mental health [36]. In the study by Pasek et al. on the protective buffering support scale, the sense of support was statistically significantly higher in caregivers than in patients [31].

The review found that research on social support in dyads is time-consuming and takes up to several years. Recruiting patients and their relatives does not seem to be an easy process. Twelve articles described cross-sectional projects. However, longitudinal studies can be planned in the future to determine the level of social support at various stages of cancer treatment. Depending on health needs, prognosis, professional and social situation, the changing picture could indicate what kind of support and at what stage is most desirable. It would then be important to know the reasons for underestimating the identification of received support and the dynamics of these changes.

This scoping review has some limitations that need to be acknowledged. Firstly, a large number of records were excluded during the screening phase, which might have led to the exclusion of some potentially relevant studies. However, the broad definition of cancer and the absence of a direct definition of a dyad in the screened titles and abstracts posed challenges in the search process, justifying the need to exclude several identified records in the screening process. Secondly, this scoping review did not include statistical pooling of effects or risk of bias assessment of the included studies, which might have limited the interpretation of the results, implying the need for future reviews aimed at describing the associations between the quality of the dyadic relationships and some patient-related outcomes, including a comprehensive assessment of the risk of bias of the primary research. Nonetheless, a scoping review focuses on mapping and summarising the available evidence rather than assessing the quality of individual studies. Finally, there is a possibility of publication bias, as there may be a lack of research in this regard, and it is reasonable to assume that some relevant studies may be in the grey literature or unpublished. Despite these limitations, the scoping review provides an overview of the recent state of knowledge on the impact of social support in a patient-informal caregiver relationship during cancer treatment, highlighting the need for further research in this area, particularly, longitudinal studies examining the changing nature of social support over time. It is important to note that scoping reviews serve a distinct purpose in the research process compared to the traditional systematic review. While scoping reviews do not provide a comprehensive synthesis with an evaluation of the risk of bias of all available literature on a topic, they do offer a valuable literature mapping and can help to identify areas where more rigorous systematic reviews may be necessary, thereby paving the way for future research.

## 5. Conclusions

The conducted scoping review highlights the significant role of social support in the dyad of an oncological patient undergoing cancer treatment and their caregiver. Given the importance of caregivers in the oncological pathway of cancer patients, it is critical to provide them with the support to overcome difficulties related to stress, fear, and threats, thus mitigating the negative effects of cancer treatment. Our findings suggest that a dyad-based approach could become a key element of oncology care, with caregivers being indispensable as both providers and recipients of care. Social support activities focused on the dyad could be an important element in the development of care strategies by national institutions. Importantly, our review highlights that social support, including its various possible approaches, works best in dyads and is associated with improved quality of life, hope, and resilience. However, we also found that support interventions received by patients, especially caregivers, were often inadequately assessed and/or described, emphasising the need for better measurement of the need for support and appropriate delivery of support interventions to the dyad. Future research should consider the various stages of the oncological pathway, from diagnosis to healing, and plan studies with larger samples and specific outcomes to more accurately assess the impact of social support interventions on dyads.

## Figures and Tables

**Table 1:** Summary of included studies.

Author/Year	Context (Study Site)	Project (Design)	Aim of Study	Tools	Group of Subjects		Statistical Analysis
					Patients	Caregivers	
Ayik et al. [23] 2022	Patients hospitalised in the oncology (medical and radiation oncology) clinic in a university hospital in eastern Turkey	A cross-sectional design	To find out the interrelation between the quality of life and social support of cancer patients and caregivers.	Patient and Caregiver Identification Questionnaire, Multidimensional Scale of Perceived Social Support, Rolls Royce Quality of Life Scale	318	318	Descriptive statistics, the Kruskal–Wallis, the ANOVA, and the Pearson’s correlation test; the level of error was accepted as a *p*-value of <0.05.
Boeding et al. [24] 2014	Couples were recruited from two major medical centres in the context of a larger treatment-outcome study. USA	A cross-sectional design	To examine the ways in which a woman’s daily mood, pain, fatigue, and her spouse’s marital satisfaction predict the woman’s report of partner support in the context of breast cancer.	Source-Specific Social Provisions Scale, Positive and Negative Affect Schedule, Brief Pain Inventory, Brief Fatigue Inventory, Baseline Measure: Quality of Marriage Index	158	159	Multilevel modelling (MLM) was used following the guidelines put forth by Raudenbush and Bryk (2002) in order to evaluate the effects of women’s pain, fatigue, and mood and men’s marital satisfaction on the amount of social support provided to women with breast cancer over a span of 30 days.
Chen et al. [25] 2021	First Affiliated Hospital of Anhui Medical University and the First Affiliated Hospital of University of Science and Technology of China in Anhui province.	A cross-sectional design	To examine the impact of family resilience on the individual resilience of couples during cancer and explore the potential mediating role of perceived social support and the moderating role of sex in this association in cancer patient-spouse dyads.	Family Resilience Assessment Scale, Perceived Social Support Scale, Resilience Scale	272	272	Descriptive Statistics, Pearson’s correlations, the mediation of their own and their partners’ perceived social support, and the actor–partner independence mediation model (APIMeM).
Dębska et al. [26] 2017	The Centre of Oncology, M. Skłodowska-Curie Memorial Institute in Cracow and at the St. Luke Provincial Hospital in Tarnów. Poland	A cross-sectional self-inventory study	To determine the level and sources of support available for cancer patients and their close relatives.	Berlin Social Support Scales and a sociodemographic-clinical survey	193	193	Student *t*-test and ANOVA (with LSD post-hoc test), Cohen’s d-coefficients. The power and direction of associations within pairs of variables were determined on the basis of Kendall’s tau-b coefficients of linear correlation.
Goldzweig et al. [27] 2016	The Institute of Oncology at Hadassah University Hospital; patients from various geographic regions of Israel were targeted. Israel, Austria	A cross-sectional study	To assess relationships between oldest andold (minimum 86 years) patients’ perceived social support with their own and their spousal caregivers’ hope through the application of the actor–partner interdependence model (APIM).	Social support, the Cancer Perceived Agents of Social Support, Geriatric Depression Scale, the distress thermometer, the Adult Hope Scale	58	58	Paired *t*-tests after establishing an interaction between role and gender and validating significant results in multi-analysis of variance [MANOVA] overall variables. Pearson’s zero-order correlation coefficients; the SPSS (version 21.0; IBM Corp. NY, USA) software.
Hasson-Ohayon et al. [28] 2014	Hadassah University Hospital, Jerusalem, Israel. Israel, USA	A cross-sectional study	To compare the relationship between social support, hope, and depression among different age groups of women with advanced breast cancer and their healthy spouses.	Cancer perceived agents of social support, the brief symptom inventory (BSI), the Adult Hope Scale	150	150	Descriptive statistics, multivariate analysis of variance (MANOVA), and structural equation modelling (SEM).
Jeong et al. [29] 2017	Gastric cancer patients and their family caregivers who visited a university medical centre. South Korea	A cross-sectional study	To investigate the moderating role of social support on the psychological well-being of both cancer patients and family caregivers.	Duke-UNC Functional Social Support Questionnaire, the Hospital Anxiety and Depression Scale	52	36	Hierarchical multiple regression analyses.
Law et al. [30] 2018	The Radiation Oncology Department at The Canberra Hospital. Canberra, Australia	qualitative study	To gain an in-depth understanding of CRC patients’ and caregivers’ experience of social support within the cancer treatment setting.	Individual interviews	22	22	The framework approach, qualitative content analysis, consisted of five interconnected stages linked to forming a methodical and rigorous audit trail.
Pasek et al. [31] 2022	Patients were in the chemotherapy and radiotherapy wards of oncology hospitals in Poland, and their caregivers Poland	Across-sectional study	To investigate the factors influencing the multidimensional aspect of social support in a cancer patient-informal caregiver dyad.	Standardised: BSSS, POS, SSCS, TIPI, ET, SPT, the authors’ own tool for sociodemographic assessment.	170	170	Descriptive statistics, non-parametric Mann–Whitney U test, multiple regression analysis using the stepwise progressive method, analysis of the distribution of residuals.
Pasek et al. [10] 2017	Patients in the chemotherapy and radiotherapy wards of oncology hospitals in Poland and their caregivers. Poland	A cross-sectional study	To analyse interrelationships between perceived support and the SOC in caregivers, and perceived support, the SOC, and acceptance of illness in cancer patients.	Standardised: BSSS, AIS, SOC-29, the authors’ own tool for sociodemographic assessment.	80	80	Descriptive statistics, analyses using Student’s *t*-test, Cohen’s d coefficient, analysis of r-Pearson’s correlation coefficients, serial bootstrapping mediation analysis—PROCESS macro for SPSS.
Sterba et al. [32] 2017	The study was conducted at a head and neck cancer (HNC) clinic in a regional cancer centre. The interested participants nominated primary caregivers, the person they reported relying on most for cancer-related support. USA	A cross-sectional study	To examine the physical and emotional well-being and social support in newly diagnosed HNC patients and caregivers and identify sociodemographic, clinical, and behavioural risk factors associated with compromised well-being in patients and caregivers.	The Short Form Health Survey (SF-12), open-ended questions so participants could respond in their own words, the MD Anderson Symptom Inventory tool for sociodemographic assessment	72	72	Descriptive statistics, ANOVAs and Fisher’s exact tests for continuous and categorical variables, respectively (*p* < 0.05).
Surucu et al. [33] 2017	University Hospital’s Gynaecologic Oncology service in Southern Turkey.	A cross-sectional study	To analyse the level of perceived social support and hope of cancer patients and their families.	Patient Social Support Form and Family Social Support Form, the Beck Hopelessness Scale	69	69	Arithmetic average, standard deviation, Mann–Whitney U test, Kruskal–Wallis test, Spearman–Brown correlation analysis.
Uslu-Sahan [34] 2018	Patients with gynaecologic cancer and their caregivers at one university hospital in Ankara, Turkey.	A cross-sectional study	To determine whether hospitalised patients with gynaecologic cancer and their caregivers differ in feelings of hopelessness and death anxiety and how those conditions may be related to their social support.	Patient Information Form, Caregiver Information Form, the Multidimensional Perceived Social Support Scale, the Beck Hopelessness Scale, the Thorson-Powell’s Death Anxiety Scale (DAS)	200	200	Student’s *t*-test, Pearson’s correlation test, and linear regression analyses. The data were analysed using the SPSS version 20 (SPSS Inc., Chicago, IL, USA).

**Table 2:** Social support studied and key findings of the studies included in the review.

Author/Year of Publication	Type of Social Support Studied	Key Results
Ayik et al. [23]	social support	The relationship between the MSPSS and Rolls Royce Quality of Life Scale of the patients was investigated; a positively important connection was determined between perceived total social support and general quality of life subscales of general well-being, medical interaction, sexual function, physical symptoms and activity, social relations, business performance scores and the total mean scores. There was a positive and important relationship between the MSPSS subscales and the total mean scores of patients and caregivers.
Boeding et al. [24]	partner support, social support, perceived support	Results show that on days in which women reported higher levels of negative or positive mood, as well as on days they reported more pain and fatigue, they reported receiving more support. Women who, on average, reported higher levels of positive mood tended to report receiving more support than those who, on average, reported lower positive mood. However, average levels of negative mood were not associated with support. Higher average levels of fatigue but not pain was associated with higher support. Finally, women whose husbands reported higher levels of marital satisfaction reported receiving more partner support, but husbands’ marital satisfaction did not moderate the effect of women’s mood on support.
Chen et al. [25]	social support, perceived social support, practical social support, friends support, family support	The results indicated that the patients’ and their spouses’ level of family resilience was positively associated with their own individual resilience directly and indirectly by increasing their own perceived social support. The family resilience of the spouses was associated with an increase in the patients’ individual resilience only indirectly by increasing the patients’ perceived social support. The spouse-actor effects between family resilience and individual resilience differed significantly by sex.
Dębska et al. [26]	support: instrumental, emotional, informational support, perceived (emotional instrumental), received (emotional; instrumental; informational; satisfaction with support)	Cancer patients had more perceived and received social support than their caregivers. Patients identified more sources of available support than their caregivers. When the level of support was stratified according to the caregiver’s relationship with the patient, caregivers-partners, and caregivers-children presented higher levels of perceived support than caregivers-siblings and caregivers-parents. Caregivers received less support than patients from medical personnel.
Goldzweig et al. [27]	social support	Patients presented high distress levels. Among patients and spouses, perceived social support was positively correlated to their own level of hope and negatively correlated to the other’s level of hope.
Hasson-Ohayon et al. [28]	sample, emotional, cognitive and instrumental support	Older patients and spouses reported lower levels of depression than younger ones. SEM showed that social support related directly to depression among younger women and older spouses, while hope was directly related to depression among older women and younger spouses, and acted as a mediator between social support and depression.
Jeong et al. [29]	perceived support	Patients’ income and social support were related to depression and anxiety, but the interaction of income and social support was only observed for anxiety. For caregivers, no interaction effects were found. Social support decreased the negative effects of low-income status on the patients. No predictors related to patients’ health or living status explained caregivers’ depression and anxiety in Model I. When social support was entered in Model II, patients’ age had a marginally significant effect on patients’ depression; patients’ income had significant predictability of patients’ anxiety. Additionally, patients’ social support predicted patients’ anxiety, whereas caregivers’ social support explained both the depression and anxiety of caregivers. There was no dyadic effect: patients’ social support neither predicted caregivers’ outcomes, nor did caregivers’ social support predict patients’ outcomes. Patients’ depression was explained by patients’ income, and patients’ anxiety was explained by income and social support, including their interaction. For caregivers’ outcomes, no predictors related to patients’ status nor did caregivers’ social support had significant predicting power.
Law et al. [30]	social support	Three major themes emerged from the data: (a) treating the team as a source of support, highlighting the importance of connection with the treating team; (b) changes in existing social supports, encompassing issues regarding distance in interpersonal relationships as a consequence of cancer; and (c) differing dimensions of support, exploring the significance of shared experience, practical, financial, and emotional support.
Pasek et al. [31]	perceived support: available, emotional, instrumental, the need for support, seeking support currently received, protective buffering support, emotional support, instrumental support, information support, satisfaction with support	On the support scales, statistically significant differences between the examined patients and their caregivers occurred for the support currently received (*p* ≤ 0.01), emotional support (*p* ≤ 0.05), and the general level of received protective buffering support (*p* ≤ 0.001). The study showed statistically significant differences in the scales of currently received support and emotional support. In both cases, the values of support indicated by patients were higher than those indicated by caregivers. On the scale of protective buffering support, the sense of support was significantly higher in caregivers than in patients. No statistically significant differences were observed in the groups of patients and their caregivers on the scales of perceived emotional support, perceived information support, the need for support, sense of support, instrumental support and information support. There was a statistically significant correlation between information support in the group of caregivers and the need for support (0.23) and the sense of support (0.16) in the group of patients. A positive correlation was obtained, indicating that the increase in information support for carers increased the need for support and the sense of support in patients.
Pasek et al. [10]	perceived support	Perceived social support scores were high in both groups; still, the values of this parameter in cancer patients turned to be higher than in their caregivers. In both patients (t = −3.82; *p* < 0.001; d = 0.40) and their caregivers (t = −2.25; *p* = 0.027; d = 0.21), the level of perceived instrumental support was higher than the level of perceived emotional support. This points to the important role of the interaction between the patient and their close relatives as a determinant of illness acceptance.
Sterba et al. [32]	received and provided support	The most frequently approved type of support identified by both patients and caregivers was emotional support, with frequent emphasis on specific types of emotional support in the form of spiritual help and help for patients with aesthetic problems and addictions. Caregivers were also more likely than patients to commonly emphasise the provision of critical instrumental support, including help with finances, transportation to appointments, cooking and other household chores.
Surucu et al. [33]	perceived social support, emotional and material support, information support	No significant correlation was found between the perceived social support of cancer patients from their relatives and the social support the relatives think they provided for the patients. Patients’ perceived social support from their relatives is higher than what the relatives think they provide for the patients. The patients and relatives had very high levels of hope; no significant correlation was found.
Uslu-Sahan [34]	social support, emotional support, perceived social support	Patients had higher hopelessness and death anxiety compared with caregivers. Patients’ perceived social support explained 35% of the total variance in hopelessness and 28% of the variance in death anxiety; caregivers’ perceived social support explained 40% of the total variance in hopelessness and 12% of the variance in death anxiety.

## Data Availability

Not applicable.

## Supplementary Materials

The following supporting information can be downloaded at https://www.mdpi.com/article/10.3390/cancers15061754/s1, Preferred Reporting Items for Systematic reviews and Meta-Analyses extension for Scoping Reviews (PRISMA-ScR) Checklist.
